# Whole Genome Characterization of *Klebsiella* Strains in European Hedgehogs and Human Nosocomial Settings Identified Shared Sequence Types, Antimicrobial Resistance Genes and Plasmids

**DOI:** 10.1111/zph.70072

**Published:** 2026-06-15

**Authors:** Biel Garcias, Chiara Seminati, Carolin M. Kobras, Samuel K. Sheppard, Rafael A. Molina‐López, Laila Darwich

**Affiliations:** ^1^ Department Sanitat i Anatomia Animals, Veterinary Faculty Universitat Autònoma de Barcelona (UAB) Cerdanyola del Vallès Spain; ^2^ Sir William Dunn School of Pathology University of Oxford Oxford UK; ^3^ Ineos Oxford Institute for Antimicrobial Research, Department of Biology University of Oxford Oxford UK; ^4^ Centre de Fauna Salvatge de Torreferrussa, Forestal Catalana, S.A., Generalitat de Catalunya Santa Perpetua de la Mogoda Spain

## Abstract

**Introduction:**

*Klebsiella pneumoniae* is a pathogen associated with healthcare‐acquired infections and antimicrobial resistance (AMR) to beta‐lactams and carbapenems. Although wild animals are not typically exposed to antibiotics, they can harbour resistant strains. The European hedgehog ( *Erinaceus europaeus* ) is increasingly found in urban areas, where it interacts with humans and livestock. Studies have identified concerning levels of AMR in hedgehogs, including Extended‐Spectrum β‐Lactam (ESBL) and carbapenems‐resistant *Klebsiella pneumoniae* strains.

**Methods:**

This study focuses on *Klebsiella* spp. isolated in hedgehogs from urban areas, using whole‐genome sequencing (WGS). We compared these isolates with openly available strains isolated from humans in the same region with the objective to have a thorough understanding of ST, AMR gene, and plasmid overlap between human and environmental compartments.

**Results:**

High AMR gene levels, including the carbapenemase *bla*_OXA‐48_, were found in the hedgehog population. Notably, human nosocomial clones, including ST307 and ST392, globally distributed sequence types also found in wildlife, were identified in both hedgehogs and humans. The presence of conjugative plasmids, including IncFIB(K) and IncL1 types, was identified in both hedgehogs and humans, highlighting plasmid dissemination as a significant factor in AMR spread.

**Conclusions:**

Although no direct transmission from wildlife to hospital settings has been conclusively demonstrated, our findings suggest that hedgehogs may play a role in bridging environmental and healthcare environments. The study underscores the need for further investigation into multidrug‐resistant *Klebsiella* spp. and other resistant bacteria in wildlife to better understand their potential role in the dissemination of resistance genes across ecosystems.

## Impacts

- European hedgehogs living in urban areas were found to carry antibiotic‐resistant *Klebsiella pneumoniae* strains, including those strains resistant to last‐resort antibiotics used in hospitals.
- The same high‐risk bacterial lineages and resistance‐carrying plasmids were identified in both hedgehogs and humans, suggesting overlap between wildlife and human bacterial populations.
- These findings highlight hedgehogs as potential environmental reservoirs linking wildlife and healthcare‐associated AMR ecosystems.

## 1 Introduction

*Klebsiella pneumoniae* is a Gram‐negative, opportunistic pathogen belonging to the *Enterobacteriaceae* family, commonly associated with healthcare‐acquired infections, including pneumonia, urinary tract infections, and septicaemia (Effah et al. 2020). It can also be found in a wide range of hosts such as livestock, pets, wildlife, insects, plants, soil or water environments (Wyres et al. 2020).

*Klebsiella* spp. strains have shown a high level of antimicrobial resistance (AMR), with antibiotic resistant strains associated with nearly 600,000 deaths worldwide in 2019 (Murray et al. 2022). The application of genomic technologies has allowed to track the epidemiology and understand the transmission of *Klebsiella* spp. in clinical settings (Kong et al. 2021), resulting in lineages (Wyres, Wick, et al. 2019). Thus, in *K. pneumoniae* it has been possible to identify multidrug‐resistant (MDR) strains producing extended‐spectrum β‐lactamases and/or carbapenemases and, in parallel, ‘hypervirulent’ strains expressing acquired virulence factors that cause severe community‐acquired infections (Wyres et al. 2020). Clones such as ST11, ST15, ST147, ST307 or ST392 are considered part of an MDR lineage (Wyres and Holt 2016), while others such as ST23, ST65 or ST66 are hypervirulent (Hv) clones (Wyres et al. 2020). However, there is increasing evidence of combined MDR‐Hv clones causing outbreaks worldwide due to convergence in plasmids (Gu et al. 2018; Lam et al. 2019).

*Klebsiella* spp. have been isolated from wildlife, but since these animals have never been treated with antibiotics, these strains may be expected to not carry AMR genes. Nevertheless, this idea seems to be not fully true, since commensal bacteria from wild animals have been found to be a reservoir of these compounds, facilitating the horizontal transmission to bacterial pathogens (Arnold et al. 2016; Carroll et al. 2015; Swift et al. 2019). High levels of AMR genes have been found in different wildlife species such as different wild birds (Wang et al. 2023), chimpanzees (Baron et al. 2021), deer (Ballash et al. 2022) or wild boars (Bachiri et al. 2018), including extended spectrum beta‐lactamases (ESBL) such as *bla*_CTX‐M‐15_ or carbapenemases such as *bla*_OXA‐48_.

These findings together with the fact that MDR isolates that cause nosocomial outbreaks such as ST11, ST15 or ST307 have been detected in wastewater (Radisic et al. 2023), livestock (Mourão et al. 2024), companion animals (Garcia‐Fierro et al. 2022), food (Kurittu et al. 2021), and wildlife (Baron et al. 2021) could suggest that direct transmission between compartments exists. However, several one health studies from Italy (Hadjirin et al. 2021), United Kingdom (Ludden et al. 2020), Guadeloupe (Dereeper et al. 2022) or India (Jacob et al. 2024) suggested that transmission is limited.

Due to the substantial diversity among *Klebsiella* isolates within the same host, demonstrating direct transmission routes remains a challenge (Wyres et al. 2020). Moreover, since behaviour and habitats of distinct species are highly diverse, particularly within the wildlife compartment, selecting one of the species would be critical to detect the transmission. Despite this, in the studies presented before, wildlife is still treated as a uniform compartment and species are randomly selected according to availability without accounting for interspecies behaviour/habitat variability.

The European hedgehog ( *Erinaceus europaeus* ) is a widely distributed and common wild species across Europe. These small, nocturnal insectivores are characterized by their spiny coats and inhabit a diverse range of environments (Hoefer 1994). While they frequently interact with humans and livestock in rural settings, recent trends indicate an increasing presence in urban and suburban areas, including residential gardens and urban green spaces (Hubert et al. 2011). This proximity to humans has been associated with antimicrobial resistance (Mourkas et al. 2024). Moreover, earthworms, which tend to acquire antibiotics directly from soil, are one of the main components of their diet, possibly contributing to an increase in antimicrobial resistance (Darwich and Molina‐López 2023).

Recently, researchers discovered that *mecC* gene, responsible of methicillin resistant in *Staphylococcus aureus* (MRSA), associated with nosocomial outbreaks emerged from hedgehogs (Larsen et al. 2022). Most whole genome sequencing (WGS) studies are still restricted to MRSA isolates (Dierikx et al. 2023; Miller et al. 2024; Venla et al. 2023), with hardly any studies focusing on *Enterobacteriaceae*, or *Klebsiella pneumoniae* specifically. Nevertheless, the few studies that have focused on *Enterobacteriaceae* carriage in European hedgehogs, have consistently identified high abundance of AMR genes (Di Francesco et al. 2020; Garcias et al. 2021). Furthermore, in Catalonia, *Erinaceus europeus* has been found highly colonized by ESBL resistant *Klebsiella pneumoniae* (Darwich et al. 2019; Garcias et al. 2021).

Herein, we hypothesised that bacterial isolates isolated from wild hedgehogs inhabiting urban areas can be good indicators of AMR and we focused particularly on *Klebsiella* spp. To assess this, we took advantage of WGS to analyse 24 *Klebsiella* genomes from a previous study (Garcias et al. 2021) representing the WGS study focused on *Klebsiella* spp. isolated from hedgehogs. Furthermore, we compared these isolates with openly available strains isolated from humans in the same zone to have a thorough understanding of ST, AMR gene and plasmid sharing.

## 2 Material and Methods

### 2.1 Isolate Selection

Twenty‐four *Klebsiella* spp. isolates were selected from a collection of a previous study performed in the province of Barcelona between 2015 and 2019. Sample collection and isolation proceeding is described in Garcias et al. (2021). Briefly, faecal samples were collected from hedgehogs attending the Wildlife Rehabilitation Center (WRC) of Torreferrussa (Catalunya, North‐East Iberian Peninsula) using sterile swabs with an Amies transport medium (Deltalab, Barcelona, Spain), before any pharmacological or antimicrobial treatment was implemented. To detect MDR strains producing extended‐spectrum β‐lactamases, the swabs were cultured on a selective medium containing MacConkey agar (Oxoid, Basingstoke, UK) supplemented with ceftriaxone (1 mg/L), and aerobically incubated for 24 h at 37°C. Growing colonies were isolated on TSA agar in order to proceed with the bacterial identification using conventional biochemical tests (oxidase, catalase, TSI, SIM, urease, citrate, and methyl red) and the API system (bioMérieux, Marcy l'Etoile, France). After identification, strains were stored in glycerol/brain heart infusion solution at −80°C until they were defrosted by culture on TSA agar for their use. Relevant metadata is presented in Table S1.

### 2.2 DNA Extraction, Sequencing and Archiving

Twenty‐four *Klebsiella* isolates were analysed by whole genome sequencing. Genomic DNA of 22 isolates was extracted using the Maxwell RSC Cultured Cell DNA Kit (Promega) on semiautomated DNA extraction machines (Maxwell RSC Instrument, Promega), following the manufacturer's instructions. DNA was quantified using the QuantiFluor ONE dsDNA System and a Quantus Fluorometer (Promega) according to the manufacturer's instructions. Isolate genomes were sequenced at the Quadram Institute using their Illumina DNA Prep (M) Tagmentation kit. Sequencing was performed using an Illumina MiSeq platform. The pooled libraries were loaded into a MiSeq system and sequenced using a MiSeq reagent kit v2 with 2× 150 cycles (Illumina Inc.). The resulting short reads were filtered, trimmed, and adapted sequences removed using Trimmomatic (default parameters, version 0.39) (Bolger et al. 2014), and SPAdes (default settings, version 3.7) (Prjibelski et al. 2020) was used for assembly purposes. Quality control was performed with FASTQC (Andrews 2010), and all assembled genomes with an estimated average sequencing depth greater than 25× and N50 above 20,000 bp were kept. Information about quality control stats is available at Table S1.

Due to the high relevance of IncFIB(K) plasmid, two additional strains were sequenced using the Oxford Nanopore Technologies (ONT) platform, specifically the MinION device equipped with R9.4.1 flow cells. Library preparation followed the standard ONT protocol for genomic DNA, and raw data were processed using Guppy v6.5.7 for base calling, resulting in high‐quality fastq files. The sequencing data were analysed using a specialized bioinformatics workflow. Genome assembly was performed with Hybracter v1.2.0 (Bouras et al. 2024) pipeline.

### 2.3 Human Context Collection

For comparison with nosocomial bacteria from the same zone, 49 *Klebsiella pneumoniae* genomes from the Barcelona province were downloaded from the IncreDBble database (Alioto et al. 2023). Briefly, this dataset is composed of high‐quality hybrid carbapenemase‐producing *Enterobacteriaceae* assemblies isolated in Spanish hospitals. For our study, we downloaded all the *Klebsiella pneumoniae* genomes isolated from 5 Catalonian hospitals (*n* = 49). Metadata can be consulted in Table S1.

### 2.4 Bioinformatic Analysis

#### 2.4.1 Sequence Typing and AMR and Virulence Genes Screening

AMR genes presence was screened using Abricate (version 1.0.0) (https://github.com/tseemann/abricate) using the Resfinder database (Florensa et al. 2022). A positive hit was considered when a gene had > 80% nucleotide identity over > 80% of the sequence length. Chromosomal point mutations conferring resistance to antibiotics and virulence genes were screened with Kleborate (Lam et al. 2021). *Klebsiella* genomes were assigned sequence types (ST) with BIGSDB‐Pasteur (https://bigsdb.pasteur.fr/klebsiella/, accessed on 18th December 2024) and confirmed with Kleborate (Lam et al. 2021).

#### 2.4.2 Plasmid Analysis

We employed a combined approach to determine the genomic location of AMR genes, classifying them as either plasmid‐ or chromosome‐associated. Initially, genomes were analysed using the plasmidEC tool (Paganini et al. 2024), an ensemble classifier that integrates outputs from Platon (Schwengers et al. 2020), RFPlasmid (van der Graaf‐Van Bloois et al. 2021), and Centrifuge (Kim et al. 2016) to differentiate contigs into chromosomal or plasmid origin. Contigs identified as plasmid‐associated were further processed with the MOB‐Suite software using the MOB‐Recon module to reconstruct potential plasmid sequences (Robertson and Nash 2018). MOB‐Suite leverages Mash and BLAST databases to identify and assemble plasmid contigs. This integrative method addresses limitations of single‐platform analyses; for example, MOB‐Suite alone has been shown to overestimate AMR gene localization on the chromosome. The inclusion of plasmidEC mitigates such misclassification by reducing chromosomal contamination. Subsequently, contigs assigned to either the chromosome or plasmids were screened using Resfinder (Florensa et al. 2022) and typed using the PlasmidFinder database (Carattoli and Hasman 2020).

#### 2.4.3 Phylogenetic Analysis

Assemblies were annotated with Prokka (version 1.14.5, using default parameters) (Seemann 2014). A pangenome specifically for the hedgehog isolates was built using Roary (version 3.13.0) (Page et al. 2015). Genes were considered part of the core genome when they were present in 95% of the isolates, resulting in 1175 genes from *Klebsiella* spp. strains. A multiple sequence alignment was built from these concatenated sequences using MAFFT (version 7.525 with default parameters) (Katoh et al. 2018). The subsequent alignment was used as input for constructing a maximum‐likelihood phylogeny using RAxML version 8 (Stamatakis 2014) with GTR‐GAMMA substitution. The resulting tree was visualized with Microreact (Argimón et al. 2016) and can be accessed online with the following link (https://microreact.org/project/mF63vvodmMTSsUAghJhUMc‐klebsiellawildlifebarcelona).

The same process was followed for 68 *K. pneumoniae* strains (19 from hedgehogs and 49 from humans). The core genome consisted of 4045 genes and interactive visualization can be consulted here: https://microreact.org/project/bun8Z2CQqJVqF4iAndc49X‐klebsiellawithhumansbarcelona.

The evaluation of strain sharing was performed for STs shared between hedgehogs and humans. In these cases, Prokka annotation generated before was used to build an ST‐specific pangenome with Roary (version 3.13.0) (Page et al. 2015). The core genome alignment (genes present in 95% of the isolates) was used as input for SNPsites (Page et al. 2015). It was considered the same strain when the SNP distance was smaller than 100 (Watt et al. 2025).

### 2.5 Data Visualization

**FIGURE 1:**
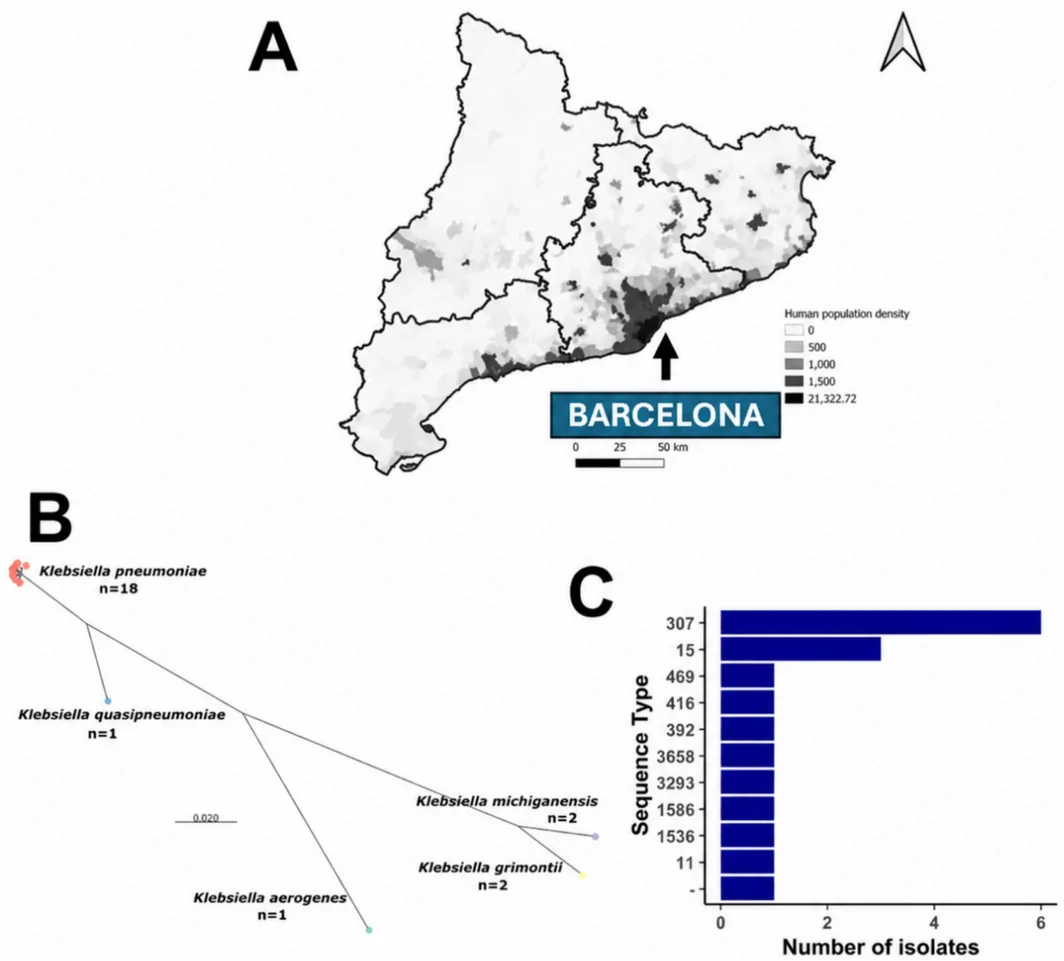
General description of the zone and the bacterial isolates. (A) Catalonian map showing the population density of Catalonia, modified from Garcias et al. (2021) to show the presence of Barcelona. Arrow points to the Barcelona province. (B) Maximum‐likelihood phylogenetic tree of 24 *Klebsiella* spp. strains isolated from hedgehogs based on the core genome (1175 genes) showing the frequency of the distinct bacterial species. (C) Barplot showing the frequency of the distinct Sequence Types (ST) of *Klebsiella pneumoniae* strains.

Apart from the already mentioned visualization of the phylogenies with Microreact, R (version 4.4.2) was used to build all other figures using the following packages: dplyr, tidyr, ggplot2, complexheatmaps and polychrome. The population density map shown in Figure 1A was created with Quantum Gis (version 3.18) (QGIS Development Team 2024). Figures were manually compounded using Inkscape.

### 2.6 Ethical Considerations

Sampling methods and animal handling techniques agreed with the Catalan Wildlife Service, which specifies the management protocols and the Ethical Principles according to Spanish legislation of the Ministry of Presidency of Spain (Spanish R.D.1201/2005).

## 3 Results

### 3.1 Nosocomial *Klebsiella* spp. Clones Are Present in Hedgehog Population

High levels of AMR and presence of ESBL and carbapenemases genes were found in *Enterobacteriaceae* isolated from hedgehogs in previous studies (Darwich et al. 2019; Garcias et al. 2021). Thus, twenty‐four *Klebsiella* spp. strains were selected for WGS to study the presence of AMR genes and their genomic context. The isolates were obtained, mostly during 2019 (*n* = 19), but some of them were retrieved in 2015 (*n* = 1), 2017 (*n* = 2) and 2020 (*n* = 2), from the Barcelona province, the zone with the highest human population density in Catalonia (Figure 1A). Most of the isolates belong to *Klebsiella pneumoniae* species complex, with *Klebsiella pneumoniae* (*n* = 18) the most common, but other species such *Klebsiella quasipneumoniae* (*n* = 1), *Klebsiella aerogenes* (*n* = 1), *Klebsiella grimontii* (*n* = 2) or *Klebsiella michiganensis* (*n* = 2) were also found in the hedgehog population (Figure 1B). Furthermore, the sequence type (ST) of *K. pneumoniae* isolates was determined, identifying ST responsible for nosocomial outbreaks such as ST307 (*n* = 6, 31.6%), ST15 (*n* = 3, 15.3%), ST392 (*n* = 1, 5.2%) or ST11 (*n* = 1, 5.2%) (Wyres and Holt 2016) (Figure 1C).

### 3.2 *Klebsiella* spp. Strains From Hedgehogs Were Mostly Multidrug Resistant but Carried Few Virulence Genes

**FIGURE 2:**
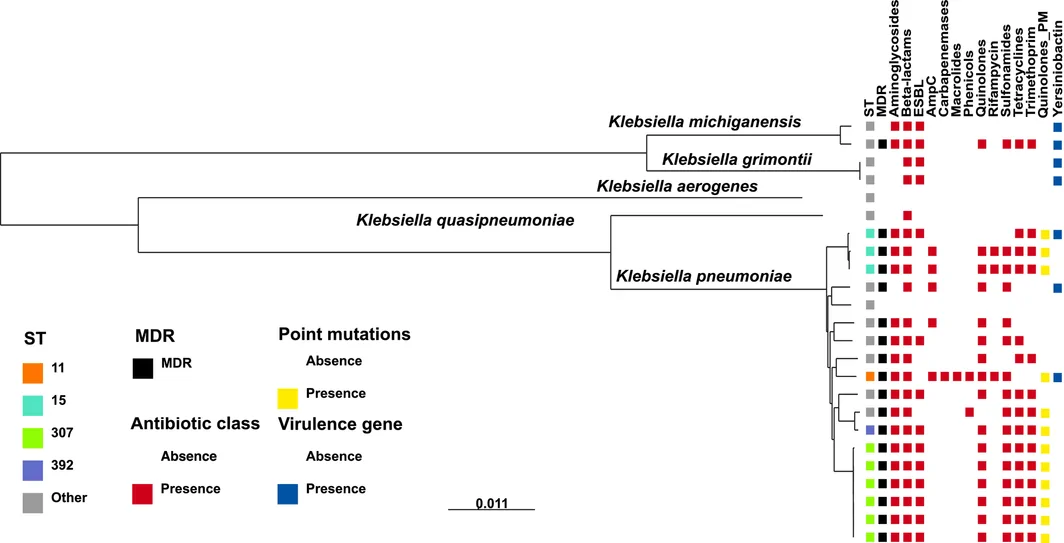
Maximum‐likelihood phylogenetic tree of 24 *Klebsiella* spp. strains isolated from hedgehogs based on the core genome (1175 genes) showing presence of STs implicated in nosocomial outbreaks (ST11: orange, ST15: light blue, ST307: blue, rest of ST: grey), MDR (black), acquired resistance genes belonging to the distinct antibiotic classes (red), chromosomal point mutations which confer antimicrobial resistance (yellow) and virulence genes (blue). *Klebsiella* chromosomal AMR genes (*bla*_*SHV*_, *fosA* and *oqxAB*) were excluded from the analysis to only show acquired antibiotic resistance determinants.

Isolates were scanned against Resfinder database (Florensa et al. 2022), a curated list of acquired AMR genes of clinical importance. A total of 41 distinct AMR genes were detected, with each *Klebsiella* isolate containing an average of 10.8 genes (range 3–16). As expected, *oqxAB* (quinolone resistance), *bla*_SHV_ (beta‐lactams), and *fos* genes were found in almost all the isolates, since they are considered part of the core genome. Considering this and that their presence does not always confer resistance (Babini and Livermore 2000; Bialek‐Davenet et al. 2015; Klontz et al. 2017), these genes were excluded from further analysis to only focus on acquired resistance. After this exclusion, we found that *Klebsiella* spp. strains carry AMR determinants conferring resistance to an average of 6 antimicrobial classes (range 0–8) (Figure 2). Notably, 75% of the strains were considered MDR (carrying three or more distinct AMR gene classes (Magiorakos et al. 2012)), with a particularly high level for *K. pneumoniae* strains (94.7%) and with only one non‐ *K. pneumoniae* isolate being MDR (*K. michiganensis*).

The most common resistance class was, as expected due to the isolation method, beta‐lactams (91.7%), but high levels were found for other commonly used antibiotic families such as aminoglycosides (75%), sulphonamides (66.7%), tetracyclines (62.5%) or trimethoprim (58.3%). Regarding families of critical importance for public health, the presence of quinolone resistance genes (different to the chromosomal *oqxAB* gene) was very frequent (70.1%), being exacerbated by the presence of quinolone point mutations present in half of the isolates. Regarding third‐generation cephalosporins, ESBL genes were also widespread across the population (58.3%), but it is also noteworthy that acquired genes encoding AmpC (20.8%) and, worryingly, carbapenemases (4.1%) were present in *Klebsiella* spp. genomes. On the other hand, the frequency of macrolide (4.1%) or phenicol (8.3%) resistance genes was low and resistance genes to a last resort option for *Enterobacteriaceae* such as colistin were not found.

Due to the existence of MDR‐Hv *K. pneumoniae* clones, isolates were also screened for virulence using Kleborate (Lam et al. 2021). The only one found was yersiniobactin, that was more prevalent in the genomes of non *K. pneumoniae* isolates and relatively rare in *K. pneumoniae* genomes (15.8%), suggesting that hedgehogs are not a reservoir of MDR‐Hv clones.

### 3.3 AMR Carriage Is Driven by Conjugative Plasmids Shared Between ST

We focused on AMR genes to have a thorough understanding of the drug resistance epidemiology (Table S2). Excluding core genome resistance genes, the most common acquired AMR determinants were the tetracycline resistance gene *tet(A)*, *aph(6)‐Id*, *aph(3″)‐Ib* (aminoglycosides), and *aac(6′)‐Ib‐cr* (aminoglycosides and quinolones), which were present in 54.2% of isolates. ESBL genes were principally represented by the presence of *bla*_CTX‐M‐15_(41.7%) and the AmpC dominant gene was *bla*_DHA‐1_. Also, the carbapenemase identified was *bla*_OXA‐48_ (4.1%), commonly implicated in nosocomial outbreaks (Guo et al. 2016).

**FIGURE 3:**
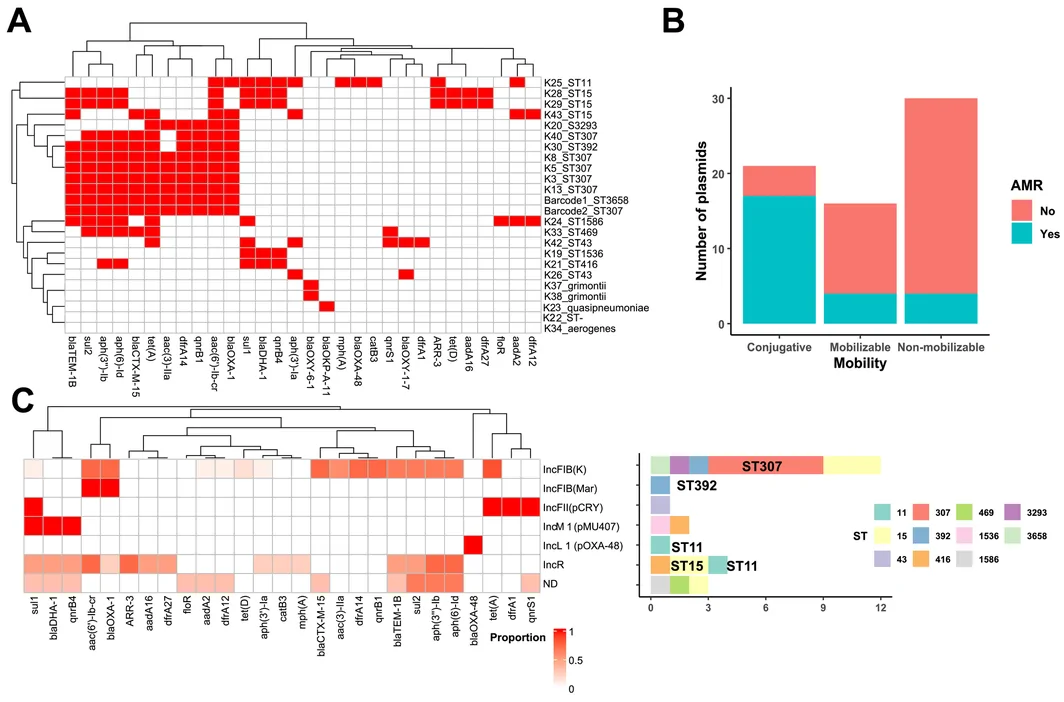
AMR gene distribution across the distinct isolates and plasmids. (A) Presence/absence dendrogram illustrating the distribution of acquired AMR genes across isolates. Isolate names are annotated with their STs. Both AMR genes (*X*‐axis) and isolates (*Y*‐axis) are hierarchically clustered using Euclidean distances. (B) Barplot showing the mobility typing of plasmids in *Klebsiella* isolates with the percentage of AMR gene carriage indicated for each group. (C) Heatmap displaying the proportion of each AMR gene in plasmids containing them. A marginal histogram shows the frequency of plasmids and their associated STs. The AMR genes (*X*‐axis) are hierarchically clustered using Euclidean distances.

A presence/absence matrix ordered by hierarchical clustering is presented in Figure 3A. A clear cluster of resistance genes is formed by isolates belonging to clinically important ST307 and ST392 (but not restricted to them since isolates with ST3293 and ST3658 were also present) containing *bla*_TEM‐1B_, *sul2, aph(3″)‐Ib, bla*_CTX‐M‐15_, *tet(A), aac(3′)‐IIa, dfrA14, qnrB1*, *aac(6′)‐Ib‐cr*, and *bla*_OXA‐1_. Moreover, other critically important genes for public health, such as *bla*_DHA‐1_ and *qnrB4*, together with *sul1*, tended to be also grouped.

Since all AMR genes, except for *bla*_OXY‐6‐1_, *bla*_OXY‐1‐7_ and *bla*_OKP‐A‐11_ (which were only present in non‐ *K. pneumoniae* isolates), were located on plasmids, we next focused on identifying the plasmid type and characteristics. Combining plasmidEC and MOB‐Suite tools, we identified 67 plasmid clusters. Each strain carried an average of 2.8 plasmids (range 0–8). According to their mobility potential, most of the plasmids were considered non‐mobilizable (45.3%), but also a great number were conjugative (31.3%). We found AMR genes on 25 plasmids (37.3%), with a clear enrichment in conjugative plasmids (17 out of 21 carried an AMR determinant), which could facilitate the dissemination of AMR genes (Figure 3B).

These plasmids were typed according to their replicon type and their frequency of carriage of AMR genes is shown in hierarchically clustered heatmap (Figure 3C). The most common plasmid type containing AMR genes was IncFIB(K) (*n* = 12), a conjugative plasmid which was mostly associated with the previously mentioned AMR cluster *bla*_TEM‐1B_‐*sul2‐aph(3″)‐Ib‐bla*_CTX‐M‐15_‐*tet(A)‐aac(3′)‐IIa‐dfrA14‐qnrB1*‐*aac(6′)‐Ib‐cr*‐*bla*_OXA‐1_, indicating acquisition of this plasmid was the main driver of MDR in these isolates. It is also important to show that the distinct replicon types were not exclusively associated with specific STs, suggesting spread between different isolates. Finally, given its nosocomial importance, it is noteworthy that *bla*_OXA‐48_ was associated with the known conjugative plasmid IncL1(pOXA‐48).

### 3.4 *Klebsiella pneumoniae* Isolates From Hedgehogs Share STs and Exhibit Similar AMR Profiles to Isolates From Humans

Hedgehogs, despite not having been treated with antibiotics, were carriers of MDR strains. Due to their urban location and behaviour, the most plausible hypothesis is that they acquired the AMR genes from anthropogenic sources. To provide genomic context, we analysed 49 *Klebsiella pneumoniae* hybrid assemblies from inCREDBle dataset (Alioto et al. 2023) isolated from human cases from the province of Barcelona. Even though it was not directly comparable (since they exclusively sequenced carbapenem‐resistant isolates), it provided a great mark to compare our newly sequenced 18 *Klebsiella pneumoniae* hedgehog strains.

**FIGURE 4:**
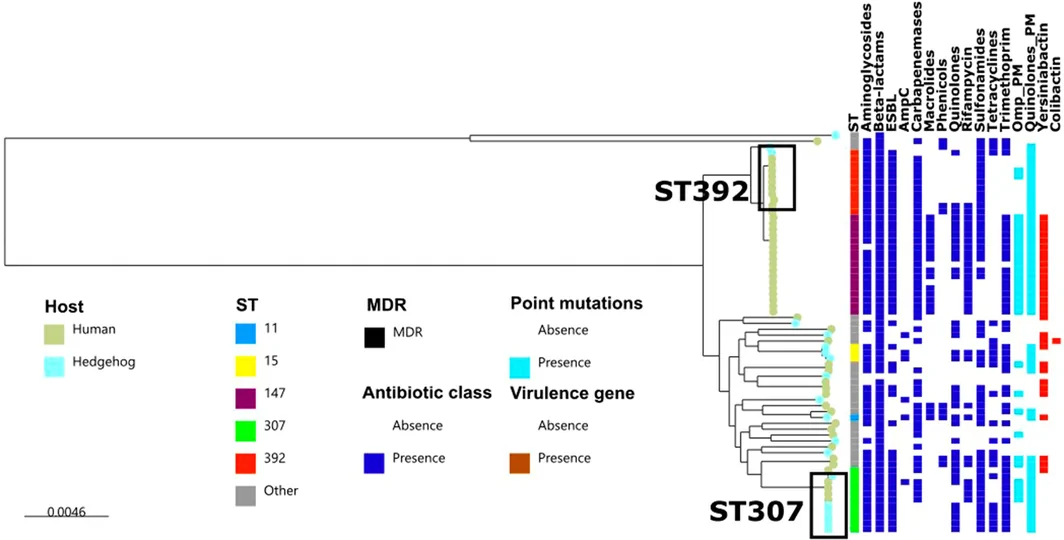
Maximum‐likelihood phylogenetic tree of 68 *Klebsiella pneumoniae* strains obtained from hedgehogs (green, *n* = 18) and human (light blue, *n* = 49) from the Barcelona province based on the core genome (4045 genes) showing presence of the most common STs implicated in global nosocomial outbreaks (ST11: orange, ST15: light blue, ST147: purple, ST307: blue, ST392: blue, rest of ST: grey), acquired resistance genes belonging to the distinct antibiotic classes (red), chromosomal point mutations which confer antimicrobial resistance (yellow) and virulence genes (blue). Shared STs between human and hedgehogs are labelled and highlighted with a rectangle. *Klebsiella* chromosomal AMR genes (*bla*_*SHV*_, *fosA* and *oqxAB*) were excluded of the analysis to only show acquired antibiotic classes.

A phylogenetic tree (Figure 4) demonstrates overlapping populations, with the clinically important MDR lineage ST392 and ST307 found in both groups, but no direct transmission could be confirmed (Tables S3 and S4). ST147 nosocomial isolates were only found in the human population. In contrast, surprisingly, ST11 and ST15, which are associated with nosocomial infections, were only found in the hedgehog population.

Comparison of the frequency of antibiotic classes is shown in Figure S1. Due to the selection bias, carbapenemases and OmpK mutations (which conferred resistance to carbapenems) were much more frequent in human isolates, but the rest of the isolates showed a similar pattern. Noticeable differences were the major carriage of macrolide resistance genes (human: 34.7% vs. hedgehogs: 5.2%) in nosocomial isolates or the major carriage of tetracycline (14.3% vs. 73.7%) and quinolones (42.9% vs. 78.9%) in hedgehog isolates. Also, it was noteworthy that, despite the total of genes conferring resistance to third‐generation cephalosporins being similar in both hosts, humans tended to acquire it more commonly via ESBL acquisition (79.6% vs. 52.7%), while hedgehogs acquired AmpC genes in a higher proportion (2.04% vs. 26.3%). Finally, although isolates from both groups could not be considered hypervirulent, human assemblies contained a higher proportion of yersiniobactin (55.1% vs. 15.8%), and colibactin was detected in one isolate.

### 3.5 Hedgehog Isolates Shared AMR Genes and Plasmids With Those Isolated From Humans

**FIGURE 5:**
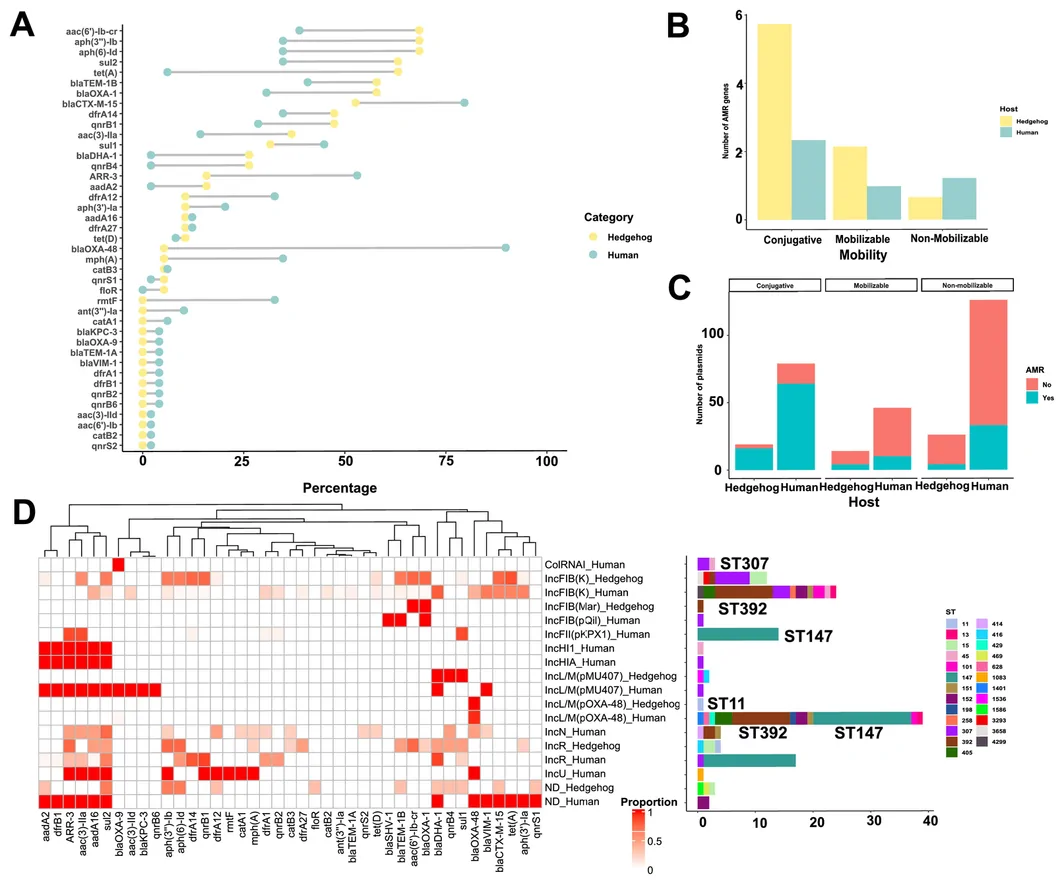
Comparison of AMR genes and plasmids between hedgehog and human isolates. (A) Cleveland dot plot comparing the frequency of AMR genes in hedgehog (yellow) and human (light blue) isolates ordered by frequency in hedgehog population. (B) Bar plot comparing the mean number of AMR genes carried by plasmids, categorized by mobility potential, between hedgehog (yellow) and human (light blue) isolates. (C) Bar plot comparing the mobility typing of plasmids in *K. pneumoniae* isolates with the percentage of AMR gene carriage indicated for each group between hedgehog (yellow) and human (light blue) isolates. (D) Dendrogram illustrating the proportion of AMR genes in plasmids shared among hedgehog and human isolates. A marginal histogram displays plasmid frequencies and their associated STs. AMR genes (*X*‐axis) are hierarchically clustered using Euclidean distances.

A more detailed comparison of hedgehog and human isolates focusing on specific AMR genes and plasmids is presented in Figure 5. Genes were commonly shared between both hosts, with those exclusively present in human isolates at a low frequency (Figure 5A). The exception was *rmtF*, which confers resistance to all available aminoglycosides, which was not found in hedgehogs but present in 30.8% of human isolates (associated with ST147). Regarding carbapenemases, the most common in human isolates was *bla*_OXA‐48_ (the one found in hedgehogs), but also *bla*_KPC‐3_ and *bla*_VIM‐1_ could be detected.

The differences between classes shown in Figure S1 are better understood in Figure 5A. For example, the higher proportion of macrolide resistance genes in humans is explained by the higher prevalence of the *mph(A)* gene. On the other hand, hedgehog isolates are assumed to have higher levels of quinolone resistance, due to the presence of *aac(6′)‐Ib‐cr* and *qnrB4*, which, at the same time, was associated with *bla*_DHA‐1_ in the same plasmid, explaining the higher prevalence of AmpC class. Finally, higher tetracycline resistance levels in hedgehogs were due to the *tet(A)* gene.

The next step was to compare plasmid from both populations. Human isolates carried a higher number of plasmids than hedgehogs (5.1 vs. 2.8), with a similar proportion of conjugative plasmids (31.5% vs. 32.2%). However, they differed in AMR carriage, because, even though overall prevalence of plasmids containing AMR genes and their mobility type was similar (Figure 5B), conjugative plasmid were more important for AMR transmission in hedgehogs since they carried a higher average number of AMR genes than those isolated from human (2.3 vs. 5.7), while non‐mobilizable plasmids carried a higher number in humans (1.2 vs. 0.7) (Figure 5C).

Finally, AMR genes present in each plasmid were compared (Figure 5D). All replicon types found in hedgehogs (except for IncFIB(Mar) (*n* = 1)) were also found in humans and, in both populations, distributed across distinct STs. However, their AMR backbone typically differed between both populations. For example, IncFIB(K) plasmids contained less and different AMR genes in humans. However, interestingly, they were the main carrier of important ESBL gene *bla*_CTX‐M‐15_.

Human isolates carried the pan aminoglycoside resistance *rmtF* gene. This was located exclusively in ST147 isolates and located in the IncFII(pKPX) plasmid, which was not found in hedgehogs, together with *aac*(3)‐IIa and ARR‐3. These isolates were especially worrying because most of them also contained *bla*_*OXA‐48*_ genes in IncL1 plasmids.

Hedgehog samples were selected using third‐generation cephalosporin resistance (CRO‐enriched MacConkey medium), whereas human samples were selected using a carbapenem‐specific medium. Despite this difference, the detection of *bla*_OXA‐48_ in a hedgehog sample makes the comparison relevant. This carbapenemase gene was located on an IncL1 plasmid, the most common plasmid type among human isolates. Although it was found in different bacterial isolates, this suggests a possible shared origin. In a more comparable population, plasmid backbone similarities would likely be more frequent.

## 4 Discussion

Hedgehogs, which inhabit urban areas and frequently are in contact with humans, have been identified as natural reservoirs of the MRSA *mecC* gene, highlighting their potential as bioindicators of AMR environmental pollution (Bengtsson et al. 2017; Larsen et al. 2022; Venla et al. 2023). However, despite their significant role as AMR sentinels, little is known about their involvement in carrying multidrug‐resistant (MDR) Enterobacteriaceae. To the best of our knowledge, this study is the first to use whole‐genome sequencing (WGS) to characterise *Klebsiella* spp. strains isolated from wild hedgehogs.

Finding resistant bacteria in wildlife is not uncommon (Quintelas et al. 2024), and Spain is no exception (Ahlstrom et al. 2022). However, most studies focus on animals like seagulls, which are well‐established AMR sentinels but require capture, causing stress to the animals and increasing workload. In this study, we propose hedgehogs admitted to wildlife rehabilitation centres as a convenient, non‐invasive sampling option for detecting AMR in wildlife. This approach could help optimise future AMR surveillance efforts, although it needs to be more explored.

Since the sharing of MDR *Klebsiella* STs between livestock and humans appears to be limited (Kaspersen et al. 2023; Ludden et al. 2020; Thorpe et al. 2022), transmission seems to be more common between companion animals (Garcia‐Fierro et al. 2022) or through wastewater (Radisic et al. 2023). These recent studies principally found typical nosocomial STs such as ST11, ST15, and ST307 in the previously mentioned compartments. These STs were also found in the present study. Transmission of MDR bacteria between wildlife and hospital environments has not been conclusively demonstrated (Thorpe et al. 2022). However, one challenge in assessing this risk is that wildlife is often treated as a single, uniform group, even though different species occupy distinct ecological niches and exhibit varied behaviours. These differences can influence their exposure to human‐associated environments. In this study, MDR was predicted in silico and no phenotypic test was performed, but our findings suggest that hedgehogs arriving at WRC could provide an accessible and practical sampling opportunity to investigate potential links between environmental reservoirs and nosocomial infections.

ST307 was the most common clone distributed worldwide (Wyres, Hawkey, et al. 2019), which already has been detected in wild animals such chimpanzees (Baron et al. 2021) or urban rats (Schaufler et al. 2018). In this study, we identified this ST in both humans and hedgehogs, carrying the typical conjugative plasmid IncFIB(K), which has been previously recognized as a key driver of MDR (Wyres, Hawkey, et al. 2019). The point here was to show how plasmids were also distributed across distinct STs in wild populations, emphasizing that plasmids should be the focus for controlling AMR dissemination. However, to gain a deeper understanding of this process, further studies utilizing long‐read sequencing techniques are necessary. On the other hand, both ST11 and ST15 have been described the most common clones isolated from nosocomial infections in Spain (Alioto et al. 2023; Salamanca‐Rivera et al. 2024). Although these strains were found in hedgehog populations, they were not detected in the human population of Barcelona. While it is possible that these STs are simply absent in the city, given that our data came from a limited sample of distinct hospitals, the lack of detection could also be due to a sampling bias rather than their true absence in the Spanish population. Additionally, we cannot rule out the possibility that these hedgehog strains originated from other sources, such as companion animals, where similar strains have been reported in other countries (Garcia‐Fierro et al. 2022). However, this former explanation seems less likely due to the low prevalence of carbapenem‐resistant strains in companion animals in Spain compared to the human frequency at nosocomial settings (Li et al. 2021).

Concretely, the ST11 clone from a hedgehog contained the *bla*_OXA‐48_ carbapenemase gene. In humans, *bla*_OXA‐48_ but also other genes like *bla*_VIM‐1_ or *bla*_KPC‐3_ have been detected, being *bla*_OXA‐48_ being the most commonly found but distributed in other STs. However, it was primarily associated with an IncL1 plasmid, the same type found in hedgehogs. This suggests two possible scenarios: (1) hedgehogs may have acquired the entire strain from the environment, but due to sampling limitations no ST11 strains were detected or (2) the plasmid itself is circulating among different clones and transferred to specific clones adapted to hedgehogs. In any case, both scenarios are concerning, as they suggest that carbapenemase genes are not limited to hospital settings but are also present in the broader environment. This increases the risk of resistance genes circulating between wildlife and human‐associated environments, potentially re‐entering healthcare settings and complicating infection control efforts.

One of the concerns of this kind of studies where samples of different origin are compared is the selection and isolation method used. Thus, wildlife strains were cultured for selecting resistance clones to 3r generation cephalosporines (CRO), while the human isolates were selected for carbapenem resistance. Adding the fact that strains were not isolated at the same moment (one year delay), all together it makes difficult to find the same clones in both compartments, as already previously suggested (Wyres et al. 2020). Due to this, we restricted the analysis to refer only ST and AMR gene and plasmid sharing and, here, we clarify that we did not have clear evidence of direct transmission between humans and hedgehogs. However, the high levels of similarity found shows significant evidence that this transmission could be a possibility. Finally, a snapshot study like that conducted in Italy (Thorpe et al. 2022) would be needed to confirm if a direct transmission phenomenon has already occurred. Nevertheless, further research is needed to determine the extent of MDR *Klebsiella* and other resistant bacteria in hedgehogs and their potential role in spreading resistance genes in the environment.

## 5 Conclusions

This study reveals that hedgehogs in Catalonia carried a high number of distinct antimicrobial resistance genes, including last resort families such as third‐generation cephalosporins, quinolones or carbapenems such as *bla*_OXA‐48_. These genes were also detected in human isolates at nosocomial settings. In addition, specific nosocomial clones like ST307 and ST392 and IncFIB(K) and IncL1 plasmid types were shared between humans and hedgehogs cohabiting the same ecosystem, suggesting that these wild animals can be a perfect target to analyse AMR environmental pollution.

This study is the first to use whole‐genome sequencing (WGS) to characterize *Klebsiella* spp. strains isolated from wild hedgehogs, proposing wildlife rehabilitation centres as a non‐invasive sampling method for detecting AMR in wildlife. This approach could enhance AMR surveillance efforts under the One Health perspective.

## Author Contributions

Conceptualization, B.G., and L.D.; Methodology, B.G., C.S., C.M.K., R.A.M.‐L., and S.K.S., Genome retrieval and bioinformatic analysis, B.G.; Statistical analysis, B.G.; Original draft preparation, B.G., and L.D.; Review and editing, B.G., C.S., R.A.M.‐L., S.K.S., and L.D.; Supervision, L.D. All of the authors have read and agreed to the published version of the manuscript.

## Funding

Biel Garcias was supported by the Departament de Recerca i Universitats de la Generalitat de Catalunya (FI‐SDUR 2020‐00376). This work was co‐funded by the European Union's Horizon Europe Project 101136346 EUPAHW.

## Conflicts of Interest

The authors declare no conflicts of interest.

## Supporting information


**Figure S1:** Bar plots comparing the frequency of the distinct antibiotic resistance gene families point mutations and virulence genes.

**Table S1:** Genomic metadata and quality control stats for *Klebsiella* strains isolated from hedgehogs and humans.

**Table S2:** Frequencies of antimicrobial resistance genes in *Klebsiella* isolates of hedgehogs.

**Table S3:** Single Nucleotide Polymorphisms (SNP) distance matrix based on the core genome alignment (5133 genes) of the isolates belonging to ST392. Assembly names starting by GCA represent isolates from humans.

**Table S4:** Single Nucleotide Polymorphisms (SNP) distance matrix based on the core genome alignment (4667 genes) of the isolates belonging to ST307. Assembly names starting by GCA represent isolates from humans.

## Data Availability

Raw reads were deposited in the GenBank database in the National Center for Biotechnology Information (NCBI) at https://www.ncbi.nlm.nih.gov/bioproject/PRJNA1464565/ under the BioProject PRJNA1464565.
