# Supplementary material for: Whole Genome Characterization of Klebsiella Strains in European Hedgehogs and Human Nosocomial Settings Identified Shared Sequence Types, Antimicrobial Resistance Genes and Plasmids
Source: Zoonoses Public Health. 2026 Jun 15;73(6):516–27. doi: 10.1111/zph.70072 (PMC13535739; doi:10.1111/zph.70072)
Supplement: Supplementary file 1 [file ZPH-73-516-s003.docx]

**Supplementary figure 1:** Bar plots comparing the frequency of the distinct antibiotic resistance gene families point mutations and virulence genes.
