# Supplementary material for: Whole Genome Characterization of Klebsiella Strains in European Hedgehogs and Human Nosocomial Settings Identified Shared Sequence Types, Antimicrobial Resistance Genes and Plasmids
Source: Zoonoses Public Health. 2026 Jun 15;73(6):516–27. doi: 10.1111/zph.70072 (PMC13535739; doi:10.1111/zph.70072)
Supplement: Supplementary file 2 [file ZPH-73-516-s005.docx]

| Gene | Percentage |
| --- | --- |
| aac(3)-IIa_1 | 29.2 |
| aac(6')-Ib-cr_1 | 54.2 |
| aadA16_1 | 8.3 |
| aadA2_1 | 12.5 |
| aph(3')-Ia_1 | 16.7 |
| aph(3'')-Ib_5 | 54.2 |
| aph(6)-Id_1 | 54.2 |
| ARR-3_4 | 12.5 |
| blaCTX-M-15_1 | 41.7 |
| blaDHA-1_1 | 20.8 |
| blaOKP-A-11_1 | 4.2 |
| blaOXA-1_1 | 45.8 |
| blaOXA-48_1 | 4.2 |
| blaOXY-1-7_7 | 8.3 |
| blaOXY-6-1_1 | 8.3 |
| blaSHV-106_1 | 37.5 |
| blaSHV-11_1 | 12.5 |
| blaSHV-145_1 | 4.2 |
| blaSHV-172_1 | 4.2 |
| blaSHV-182_1 | 4.2 |
| blaSHV-187_1 | 12.5 |
| blaTEM-1B_1 | 45.8 |
| catB3_2 | 4.2 |
| dfrA1_8 | 4.2 |
| dfrA12_8 | 8.3 |
| dfrA14_5 | 37.5 |
| dfrA27_1 | 8.3 |
| floR_2 | 4.2 |
| fosA_2 | 37.5 |
| fosA5_1 | 8.3 |
| fosA6_1 | 54.2 |
| mph(A)_2 | 4.2 |
| oqxA_1 | 100.0 |
| oqxB_1 | 100.0 |
| qnrB1_1 | 37.5 |
| qnrB4_1 | 20.8 |
| qnrS1_1 | 8.3 |
| sul1_5 | 29.2 |
| sul2_2 | 50.0 |
| tet(A)_6 | 54.2 |
| tet(D)_1 | 8.3 |

**Supplementary table 2.** Frequencies of antimicrobial resistance genes in *Klebsiella* isolates of hedgehogs.
